# Real-time polymerase chain reaction detection and surgical treatment of thoracic and lumbar spondylitis due to *Brucella* infection: two typical case reports

**DOI:** 10.3389/fpubh.2024.1396152

**Published:** 2024-05-22

**Authors:** Bo Liu, Yun-xiao Ji, Chang-song Zhao, Qiang Zhang

**Affiliations:** Department of Orthopaedics, National Center for Infectious Diseases, Beijing Ditan Hospital, Capital Medical University, Beijing, China

**Keywords:** real-time PCR, surgery treatment, foraminal endoscopy, Gram staining, *Brucellosis*, brucellosis spondylitis, case report

## Abstract

**Background:**

Spondylitis caused by *Brucella* infection is a rare but challenging condition, and its successful management depends on timely diagnosis and appropriate treatment. This study reports two typical cases of thoracic and lumbar brucellosis spondylitis, highlighting the pivotal roles of real-time polymerase chain reaction (real-time PCR) detection and surgical intervention.

**Case presentation:**

Case 1 involved a 49-year-old male shepherd who presented with a 6-month history of fever (40°C), severe chest and back pain, and 2-week limited lower limb movement with night-time exacerbation. Physical examination revealed tenderness and percussion pain over the T9 and T10 spinous processes, with grade 2 muscle strength in the lower limbs. CT showed bone destruction of the T9 and T10 vertebrae with narrowing of the intervertebral space, whereas MRI demonstrated abnormal signals in the T9–T10 vertebrae, a spinal canal abscess, and spinal cord compression. The Rose Bengal plate agglutination test was positive. Case 2 was a 59-year-old man who complained of severe thoracolumbar back pain with fever (39.0°C) and limited walking for 2 months. He had a 2.5 kg weight loss and a history of close contact with sheep. The Rose Bengal test was positive, and the MRI showed inflammatory changes in the L1 and L2 vertebrae. Diagnosis and treatment: real-time PCR confirmed *Brucella* infection in both cases. Preoperative antimicrobial therapy with doxycycline, rifampicin, and ceftazidime-sulbactam was administered for at least 2 weeks. Surgical management involved intervertebral foraminotomy-assisted debridement, decompression, internal fixation, and bone grafting under general anesthesia. Postoperative histopathological examination with HE and Gram staining further substantiated the diagnosis. Outcomes: both patients experienced significant pain relief and restored normal lower limb movement at the last follow-up (4–12 weeks) after the intervention.

**Conclusion:**

Real-time PCR detection offers valuable diagnostic insights for suspected cases of brucellosis spondylitis. Surgical treatment helps in infection control, decompression of the spinal cord, and restoration of stability, constituting a necessary and effective therapeutic approach. Prompt diagnosis and comprehensive management are crucial for favorable outcomes in such cases.

## Introduction

Brucellosis, a zoonotic disease caused by bacteria of the genus *Brucella*, is a significant public health concern worldwide, particularly in regions with a high prevalence of livestock farming and inadequate disease control measures (1, 2). While brucellosis primarily manifests as an acute febrile illness, it can also lead to chronic complications, including osteoarticular involvement, with spondylitis being one of the most debilitating forms. Among them, lumbar brucellosis spondylitis patients are more common, followed by thoracic vertebrae, and cervical spine is relatively rare (3). Spondylitis caused by *Brucella* infection, although relatively uncommon, poses a diagnostic and therapeutic challenge due to its insidious onset, non-specific clinical presentation, and potential for severe neurological deficits if left untreated. Thoracic and lumbar spondylitis are particularly concerning as they can cause spinal cord compression and consequent neurological impairment. In recent years, spinal brucellosis has shown an obvious upward trend (1), but it lacks specific diagnostic indicators, making clinical diagnosis difficult. Additionally, the treatment of spinal brucellosis remains controversial. At present, most scholars believe that the use of antibiotics and chemotherapy are still the main methods of treatment for this disease (2). Most cases of brucellosis can be cured by conservative treatment with drugs, but the treatment course is long and the patient suffers from long-term pain. However, surgical intervention is necessary in patients with progressive kyphosis, neurologic dysfunction, spinal instability, abscess formation, intractable low back pain, and failure to respond to conservative treatment (4).

Early and accurate diagnosis is crucial for initiating prompt and effective treatment. While conventional microbiological techniques, such as blood cultures and serological tests (e.g., Rose Bengal plate agglutination), aid in the initial diagnosis, molecular methods such as real-time polymerase chain reaction (real-time PCR) offer higher sensitivity and specificity, especially in chronic or relapsing cases (5). Real-time PCR is a highly sensitive and specific molecular diagnostic technique that can rapidly and accurately detect the target pathogen’s nucleic acid sequence. In the diagnosis of brucellosis spondylitis, real-time PCR can detect the specific gene sequence of *Brucella* from patients’ blood, tissue, or other samples, providing direct microbiological evidence of the infection (6).

In addition to antimicrobial therapy, surgical intervention plays a pivotal role in managing complicated cases of brucellosis spondylitis. Intervertebral foraminoscopy-assisted debridement, decompression, and stabilization procedures are often necessary to alleviate neural compression, control the infection, and restore spinal stability. Intervertebral foraminoscopy is a minimally invasive surgical technique in which a small incision is made at the patient’s intervertebral foramen, and an approximately 8 mm diameter operating sheath and endoscope are inserted under imaging guidance to perform debridement, decompression, and bone grafting procedures (7, 8). Compared to traditional open surgery, intervertebral foraminoscopy is less traumatic, involves less blood loss, and allows for faster postoperative recovery. Simultaneously, it can effectively remove the infected lesion, decompress the nerve roots and spinal cord, perform internal fixation, and enable bone grafting, thereby effectively controlling the infection, relieving neural compression, and restoring spinal stability (4).

This case report aims to highlight two typical cases of thoracic and lumbar brucellosis spondylitis encountered in our hospital, emphasizing the diagnostic utility of real-time PCR and the crucial role of surgical management, particularly intervertebral foraminoscopy-assisted debridement, in achieving favorable outcomes. Furthermore, it underscores the importance of histopathological examination, including Gram staining, in confirming the diagnosis and guiding appropriate treatment strategies. By presenting these cases, we aim to raise awareness among clinicians about the challenges posed by brucellosis spondylitis and the potential benefits of a multidisciplinary approach combining molecular diagnostics and surgical intervention for the successful management of this debilitating condition.

## Case presentation

### Case 1

**Figure 1:**
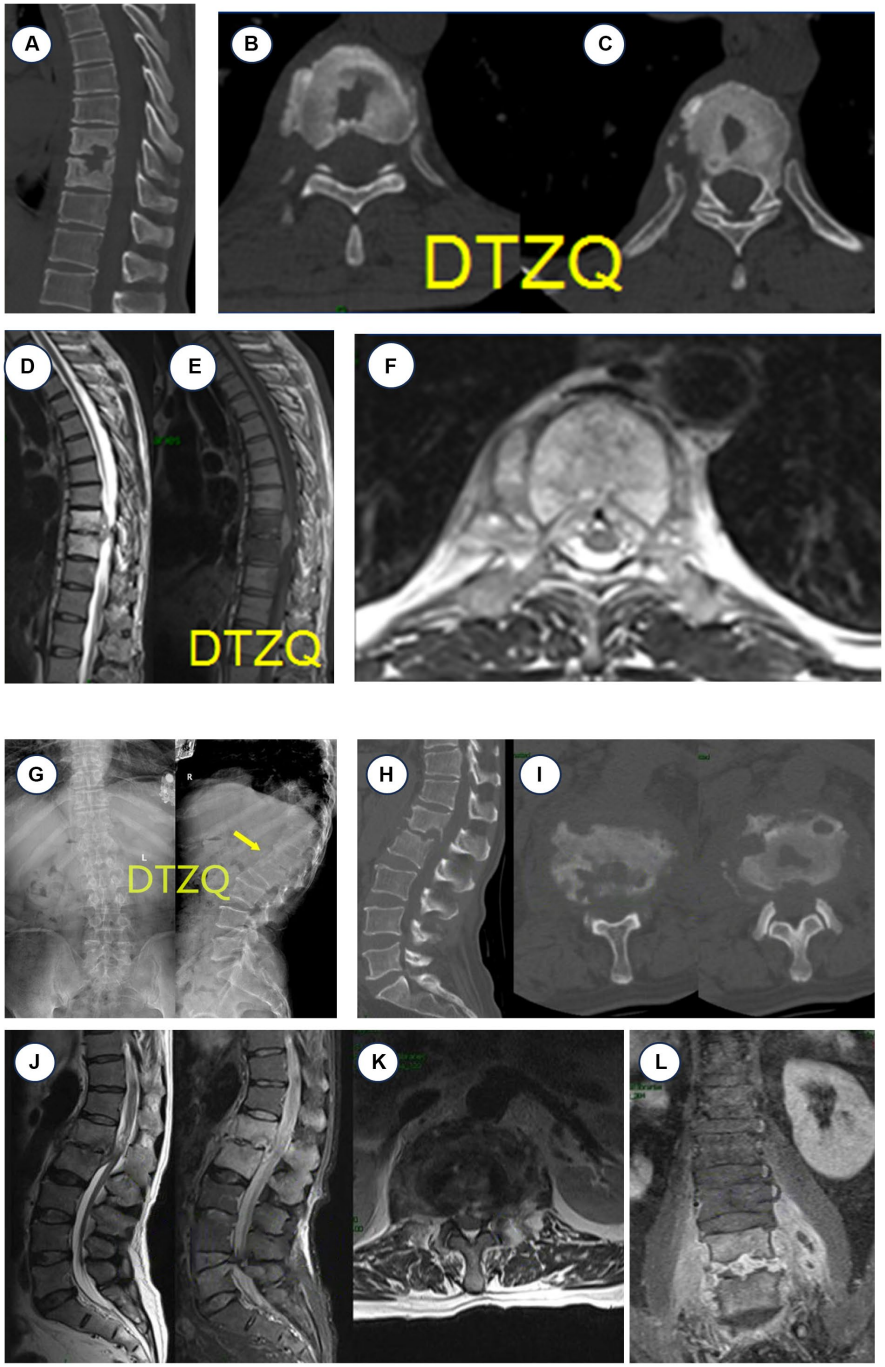
Case 1 CT and MRI manifestations of thoracic brucellosis spondylitis (T9–T10). **(A–C)** CT: T9, T10 vertebral bone destruction, intervertebral space narrowing. **(D–F)** MRI: T9–T10 vertebral body signal abnormality, vertebral body destruction with abscess, thoracic spinal cord compression. Case 2 X-ray, CT, and MRI findings of brucellosis spondylitis. **(G)** X-ray L1–L2 intervertebral space significantly narrowed. **(H,I)** CT showed worm-like bone destruction at L1–L2. **(J–L)** MRI showed inflammatory changes in the disk and vertebral body at L1–L2, abnormal high signal intensity, narrowing of the disk space, abscess formation, inflammatory tissue protruding into the spinal canal, and spinal cord compression.

A 49-year-old male shepherd presented at our hospital with complaints of severe chest and back pain persisting for 6 months. He also reported a recent onset of lower limb mobility limitation over the past 2 weeks, which notably worsened at night. His fevers spiked to 39°C. His professional background involved prolonged exposure to sheep, which was particularly relevant given our initial diagnostic considerations. Upon examination, he displayed significant tenderness and percussion pain at the T9 and T10 spinous processes. His neurological assessment revealed a muscle strength of grade 2 in both lower limbs, highlighting substantial functional impairment. Initial laboratory tests indicated an elevated white blood cell count of 11.5 × 10^9^/L with 67% neutrophils, an erythrocyte sedimentation rate (ESR) of 10 mm/h, and a C-reactive protein (CRP) level of 9.8 mg/L, indicating the presence of inflammation. A positive result on the Rose Bengal plate agglutination test strongly suggested an infection with *Brucella*. Comprehensive imaging was undertaken to further evaluate the extent of the infection. Thoracic CT scans revealed considerable bone destruction and narrowing of the intervertebral spaces at T9 and T10, with local endplate destruction evident (Figures 1A–C). MRI scans demonstrated degenerative changes in the intervertebral disks and abnormal high signal changes on T2-weighted images (T2WI), indicative of an abscess compressing the spinal cord (Figures 1D–F).

**Figure 2:**
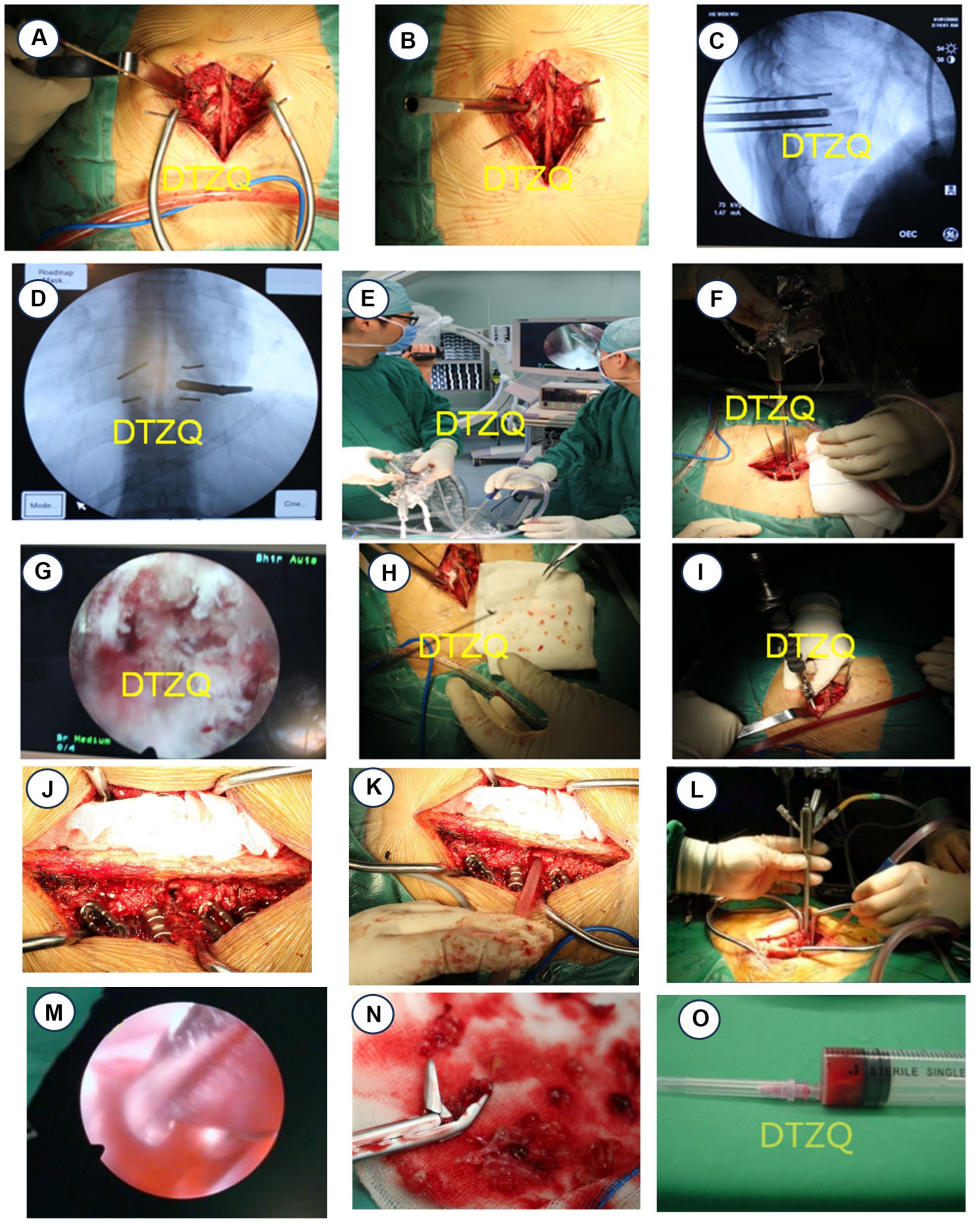
Case 1 Surgical procedure of case 1 with thoracic brucellosis. **(A,B)** Positioning pins were placed in T9 and T10 vertebral bodies. **(C,D)** Intervertebral foraminal sleeve was placed in T9 and T10 intervertebral spaces under C-arm fluoroscopy. **(E–G)** Lesions were cleared under intervertebral foraminal scope. **(H)** Abscess lesions were cleared and sent to pathology. **(I)** Pedicle screws T9 and T10 were placed under direct vision, totaling 4. Case 2 Operative procedure for patient with lumbar brucellosis spondylitis. **(J,K)** A total of eight pedicle screws were placed at T12, L1, L2, and L3, and an intervertebral foraminal sleeve was placed at L1–L2. **(L,M)** Focus was cleared under the intervertebral foraminoscope. **(N)** The abscess focus was cleared and sent to pathology. **(O)** Approximately 4 mL of blood was extracted from a 20 mL empty needle for culture.

**Figure 3:**
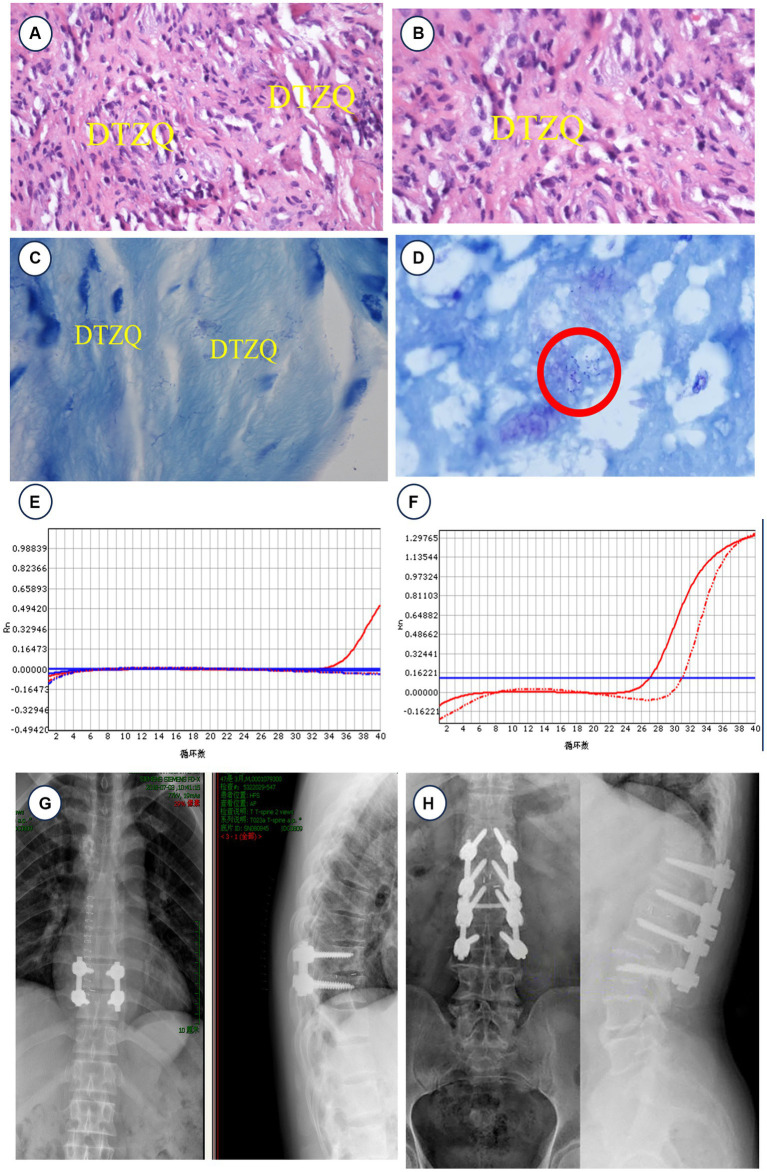
**(A,B)** HE staining showed the extensive infiltration of inflammatory cells (oil immersion, 40× magnification). **(C,D)** Gram staining of the nucleus pulposus reveals a significant number of *Brucella* (oil immersion, 100× magnification). Gram staining of the nucleus pulposus revealed the presence of small Gram-negative coccobacilli, consistent with *Brucella* organisms. **(E)** Real-time PCR of case 1 detects *Brucella melitensis* DNA at the 34th cycle. **(F)** *Brucella melitensis* and *Brucella bovis* (*Brucella* in cattle) DNA were detected in the 27th and 31st cycles of real-time PCR in case. **(G)** X-ray results of patient 1 at 4 weeks postoperatively. **(H)** X-ray of patient 2 at 12 weeks postoperatively.

Given the severity of the infection and its impact on the spinal column, we initiated an aggressive antimicrobial regimen. The patient was treated with doxycycline 200 mg daily and rifampicin 600 mg daily, supplemented with quinolones and third-generation cephalosporins, for at least 2 weeks before any surgical intervention. The surgical approach involved transforaminal-assisted posterior debridement, internal fixation, and bone graft fusion under general anesthesia. The operation was carefully documented, showing the precise placement of guide wires and the thorough debridement process using various sizes of nucleus pulposus forceps for lesion clearance (Figures 2A–I). Postoperative care included continuing the antimicrobial regimen, with added nutritional support and blood transfusions as needed. The histopathological examination with HE and Gram staining showed the extensive infiltration of inflammatory cells and confirmed *Brucella* infection (Figures 3A,C). Real-time PCR further confirmed the presence of *Brucella melitensis* DNA (Figure 3E). The patient’s recovery was notable; he was provided with a waist brace and began ambulating 1 week post-surgery. At the 12-week follow-up, he reported significant relief from back pain, and his muscle strength in the lower limbs improved to grade 3. Follow-up imaging showed stable surgical repairs and proper alignment (Figure 3G).

### Case 2

A 59-year-old man from Hulunbuir City, Inner Mongolia, presented with severe lower back pain and a fever that had persisted for 6 months, along with reduced mobility and difficulty walking that had developed over the last 2 months. His temperature had peaked at 40.1°C, accompanied by significant pain in the chest, waist, and back. A positive Rose Bengal plate test (RBPT) and an MRI revealed inflammatory changes and structural destruction in the lumbar vertebrae 1 and 2, leading to a diagnosis of brucellosis spondylitis (Figures 1G–L). The patient’s close contact with sheep in a pastoral region was a critical factor in his medical history. After 20 days of hospital treatment in a local hospital with oral doxycycline and rifampicin, along with intravenous levofloxacin, his body temperature normalized, but the severe back pain persisted. This prompted his transfer to our hospital for further diagnosis and specialized treatment. Upon admission, the patient reported poor sleep and a weight loss of approximately 2.5 kg since the onset of the disease. His physical examination revealed thoracolumbar segment tenderness, pressure pain, and radiating pain in the right lower limb, with significantly limited lumbar motion. Laboratory assays showed a leukocyte count of 4.57 × 10^9^/L with 51.30% neutrophils, a CRP of 14.9 mg/L, and an ESR of 20 mm/h. The comprehensive diagnostic workup included a negative tuberculosis antibody test and sputum smear, highlighting the specificity of the brucellosis infection.

Before surgical intervention, the patient was placed on a robust triple anti-brucellosis regimen consisting of oral doxycycline and rifampicin, supplemented by intravenous ceftazidime-sulbactam, adhering to principles of long-term, sufficient, and combined multi-route administration. Once his inflammatory markers stabilized, he underwent lumbar posterior debridement and decompression, internal fixation, and bone graft fusion under general anesthesia, facilitated by an intervertebral foramen endoscope. During the operation, the patient was positioned prone, and the surgical site was accessed through a layered skin incision. Paravertebral muscles were stripped, and a total of eight pedicle screws were inserted across T1, L1, L2, and L3. The upper and lower articular processes of L1–L2 were removed, and the intervertebral foraminal sleeve was positioned under C-arm X-ray fluoroscopy. Continuous saline irrigation was used to thoroughly remove abscess tissue, concurrently relieving nerve root compression. Diseased intervertebral disk material was meticulously cleared using a bone file and curette, with rifampicin and bone graft particles implanted into the intervertebral space to ensure stability and promote healing (Figures 2J–O). HE staining clearly shows the extensive infiltration of inflammatory cells, which is a hallmark of *Brucella* spondylitis (Figure 3B), and Gram staining revealed numerous *Brucella* (Figure 3D). The real-time PCR tests identified the DNA of both *Brucella melitensis* and *Brucella bovis* (*Brucella abortus* in cattle), confirming the dual infection (Figure 3F). Following surgery, the patient continued the anti-brucellosis medication for 6 weeks at the same preoperative dosage; 4 weeks postoperatively, the patient returned for follow-up, where X-rays and a three-dimensional CT scan of the lumbar spine showed substantial relief from pain, and he reported free movement of the lower limbs, marking a significant improvement in his quality of life (Figure 3H).

## Discussion

Our study reported two rare cases of spinal brucellosis, highlighting the key clinical features, diagnostic approaches, and comprehensive treatment involving both antimicrobial therapy and surgical intervention. In the first case, a 49-year-old shepherd presented with persistent fever, severe back pain, and lower limb weakness with ambulatory difficulties. Imaging revealed destruction of the T9–T10 intervertebral disk and an epidural abscess compressing the spinal cord. The second case involved a 59-year-old man with symptoms of fever, lumbosacral pain, and radicular leg pain. Imaging demonstrated the destruction of the L2–L3 intervertebral disk, epidural abscess, and spinal cord compression. Real-time PCR testing confirmed brucellosis in both patients. After prolonged preoperative antimicrobial therapy, they underwent decompressive surgery with instrumentation and bone grafting. Intraoperative tissue samples further substantiated the diagnosis of *Brucella* infection. Within 4–12 weeks postoperatively, both patients experienced significant alleviation of their clinical symptoms and neurological deficits. The study underscored the pivotal role of real-time PCR testing in early diagnosis of suspected spinal brucellosis cases. Once diagnosed, in addition to long-term antimicrobial therapy, surgical debridement, decompression, and reconstruction are crucial for controlling the infection, preventing complications, and improving outcomes. Timely comprehensive management was vital for a favorable prognosis in this rare but serious infectious disease.

Brucellosis is a kind of systemic zoonotic regional epidemic disease, which occurs all over the world, especially in Mediterranean and Arab regions (9–11). In China, the disease is mainly concentrated in the northern pastoral areas, which presents a certain regional aggregation (12). In recent years, the incidence of brucellosis has increased due to the increasing use of dairy products by urban and rural residents in China. Studies have shown that there are 500,000 to 1,000,000 new cases of brucellosis every year (13). The disease predominantly affects young, middle-aged, and older adults, while occurrences in infants are rare (12). Patients often present with varying degrees of contact history with cattle and sheep. Given the disease’s protracted course and propensity for spinal invasion, it can lead to a series of severe outcomes. This has resulted in increasing attention towards this disease. *Brucella* spreads through the blood, making it easy to infect the liver, spleen, lymph nodes, bone marrow, and other tissues and organs rich in mononuclear macrophages. Common clinical manifestations include low afternoon fever, weight loss, night sweats, and loss of appetite (14). In severe cases, after infection of the skeletal system, multiple migratory osteoarthralgia, myalgia, and persistent back pain were often seen throughout the body, accompanied by radiation radicular pain and spinal cord compression (3, 15). Patients’ blood inflammatory markers were elevated. The Rose Bengal plate agglutination test and tube agglutination test were common serological diagnostic methods for brucellosis. If the Rose Bengal plate agglutination test is above ++, combined with clinical symptoms and signs, brucellosis can be preliminarily diagnosed (5, 16).

Spondylitis caused by *Brucella* infection is an uncommon but challenging infectious disease. Although a clinical manifestation of brucellosis, spinal involvement is relatively rare and can be easily overlooked or misdiagnosed. The clinical presentations of brucellosis spondylitis are diverse, ranging from fever, back pain, and radicular pain to neurological deficits and motor impairment, making it difficult to distinguish from other more common spinal disorders (3). Currently, the diagnosis of brucellosis spondylitis primarily relies on serological testing and imaging modalities. However, serological methods such as the Rose Bengal plate agglutination test have limited sensitivity and specificity, and a positive result cannot differentiate between acute and chronic infections. Imaging techniques such as CT and MRI can reveal vertebral destruction, abscesses, and spinal cord compression, but these findings lack specificity and cannot definitively identify the causative agent (5, 14).

In contrast, real-time PCR detection specifically amplifies *Brucella* DNA, allowing for rapid and accurate identification of the infectious pathogen, particularly in cases with negative or indeterminate serological results (5, 6). In the two cases presented, positive real-time PCR results provided critical evidence for the diagnosis of brucellosis. Additionally, PCR testing can be applied to intraoperative tissue samples, further substantiating the diagnosis. Therefore, real-time PCR not only serves as an early diagnostic tool for brucellosis spondylitis but can also be used for monitoring treatment efficacy and follow-up. In summary, the diverse clinical manifestations and lack of specificity in conventional diagnostic tests are major contributors to the misdiagnosis or delayed diagnosis of brucellosis spondylitis. Real-time PCR plays a pivotal role in the diagnosis of this condition, facilitating timely identification of the causative agent and guiding subsequent treatment approaches.

As far as we know, there are limited literature reports on the treatment of brucellosis spondylitis at home and abroad. However, most patients can be cured by conservative treatment based on drug chemotherapy. Conservative measures include bed rest, lumbar protection, pain relief, cooling, nutritional support, and antibiotics (17). Among them, the most important is to choose sensitive and effective antibiotic drug treatment. There are different reports about which drugs should be used, but the first principle of “long-term, sufficient, combined, and multi-route administration” should be followed (18). The WHO recommended treatment regimen is as follows: doxycycline 200 mg/day and rifampicin 600–900 mg/day; for serious patients, additional quinolones and third-generation cephalosporins antibiotics may be used; and the treatment duration is typically 6 weeks (18). Furthermore, many scholars have not yet reached a consensus on the surgical treatment of brucellosis spondylitis (4, 19). According to different approaches, surgical methods could be divided into anterior debridement and bone graft fusion; posterior laminectomy, bone graft fusion, and internal fixation; anterior debridement and bone graft fusion combined with posterior internal fixation (4). The advantage of the anterior approach lies in its wide field of vision, more thorough focus clearance and nerve decompression, and lower recurrence rate. However, it is associated with potential complications such as large-vessel injury and postoperative intestinal obstruction (20), and inability to provide strong internal fixation for the diseased vertebra. Posterior approaches, though far from the abdominal cavity, avoid some serious complications (21), but Joaquim et al. (22) believe that posterior total laminectomy disrupts the normal PLC structure of the spine, leading to long-term spinal instability, kyphosis, and spondylolisthesis. Katonis et al. (4) have also reported 10 patients with brucellosis treated with percutaneous diskectomy, catheterization, anterior or posterior focal resection, bone graft, and internal fixation, with significant results.

In managing brucellosis spondylitis, a comprehensive approach that integrates medical treatment, surgical intervention, and specialized care is crucial. Initially, patients presenting with symptoms suggestive of the condition were recommended to undergo conservative medical management with antibiotics. A triple anti-brucellosis regimen, consisting of oral doxycycline and rifampicin supplemented by intravenous ceftriaxone-sulbactam, proved effective in resolving symptoms in most cases. However, close monitoring of patients was essential to identify any signs of progression or lack of response to this conservative treatment. In specific situations where conservative treatment may not have sufficed, surgical intervention became necessary. Surgical intervention was particularly indicated in cases of progressive kyphosis, neurologic dysfunction indicating spinal cord compression, spinal instability, abscess formation around the spine unresponsive to antibiotics, intractable low back pain significantly impacting the patient’s quality of life, or lack of improvement or worsening of symptoms despite appropriate antibiotic therapy. Beyond medical and surgical treatments, specialized care for patients with brucellosis spondylitis is also critical. This included regular monitoring through frequent follow-up visits to assess treatment response, symptom monitoring, and therapy adjustments as needed. Adequate pain management is essential to improve the patient’s quality of life during treatment. Nutritional support can help patients recover and enhance their immune systems, while psychological support can assist in coping with the emotional and psychological impact of the disease. Rehabilitation services, including physical therapy, can be provided to help patients regain strength and mobility, especially after surgical intervention. In conclusion, the integrated approach of medical treatment, surgical intervention, and specialized care is crucial in effectively managing brucellosis spondylitis, ensuring optimal outcomes for patients.

## Conclusion

Our study reports two typical cases of thoracic and lumbar brucellosis spondylitis, highlighting the diagnostic value of real-time PCR and the necessity of surgical intervention alongside antimicrobial therapy. It underscored the clinical significance of timely diagnosis and appropriate treatment strategies for brucellosis spondylitis, a rare but potentially debilitating condition. The diverse clinical presentations and non-specific findings on conventional diagnostic tests often lead to delayed recognition and misdiagnosis. Real-time PCR detection offers a valuable diagnostic tool, enabling early identification of the causative pathogen and facilitating prompt initiation of targeted therapy. Furthermore, the study highlights the importance of surgical intervention in conjunction with antimicrobial therapy for optimal management of brucellosis spondylitis. Surgical debridement, decompression, and stabilization are crucial for controlling the infection, relieving spinal cord compression, and restoring spinal stability, ultimately improving clinical outcomes and preventing long-term complications. In summary, prompt diagnosis through real-time PCR testing and comprehensive treatment comprising antimicrobial therapy and surgical intervention are essential for favorable prognosis in cases of brucellosis spondylitis. Early recognition and appropriate management of this rare but severe infectious disease can significantly improve patient outcomes and quality of life.

## Data availability statement

The original contributions presented in the study are included in the article/supplementary material, further inquiries can be directed to the corresponding authors.

## Ethics statement

The Ethics Committee of the Beijing Ditan Hospital of Capital Medical University approved the study. Written informed consent was obtained from the individual(s) for the publication of any potentially identifiable images or data included in this article.

## Author contributions

BL: Writing – original draft. Y-xJ: Writing – original draft. C-sZ: Writing – review & editing. QZ: Writing – review & editing.
